# E2F7 enhances hepatocellular carcinoma growth by preserving the SP1/SOX4/Anillin axis via repressing miRNA‐383‐5p transcription

**DOI:** 10.1002/mc.23454

**Published:** 2022-08-04

**Authors:** Fengjie Hao, Nan Wang, Yifan Zhang, Wen Xu, Yongjun Chen, Xiaochun Fei, Junqing Wang

**Affiliations:** ^1^ Department of General Surgery Ruijin Hospital, Shanghai Jiao Tong University School of Medicine Shanghai P.R. China; ^2^ State Key Laboratory of Bioreactor Engineering and Shanghai Key Laboratory of New Drug Design, School of Pharmacy East China University of Science and Technology Shanghai P.R. China; ^3^ Department of Pathology Ruijin Hospital, Shanghai Jiao Tong University School of Medicine Shanghai P.R. China

**Keywords:** E2F7, hepatocellular carcinoma, miR‐383‐5p, SP1/SOX/Anillin axis, tumor growth

## Abstract

E2F family participates in most human malignancies by activating the transcription of the cell cycle‐related genes. Whereas, as a specifical atypical member of this family, E2F7 was described as a repressor against its downstream genes and exerted oscillatory and controversial functions in cancers. Our previous study identified a molecular interaction promoting hepatocellular carcinoma (HCC) growth induced by SOX4 and Anillin. Meanwhile, we preliminarily identified SP1 as the upstream activator of SOX4. Intriguingly, we observed that the repressive E2F7 presents a remarkable high expression in HCC, and is positively correlated and involved in the same pathway with the potentially SP1/SOX4/Anillin axis. However, their exact interaction or mechanism controlling tumor progress between these genes has not been illustrated. Thus, we focused on this point in this study and attempted to improve the potential regulating axis in HCC cell proliferation and tumor growth for promoting tumor prevention and control. The expression profile of E2F7 in HCC tissues and tumor cells was detected along with the related candidate genes, through real‐time quantitative polymerase chain reaction assay, the Western blot analysis, and the immunohistochemistry assay, combined with bioinformatics analysis of the HCC information from the the Cancer Genome Altas and Gene Expression Omnibus data sets. The correlation between E2F7 and HCC patients' clinicopathologic features was explored. Gain‐of and loss‐of‐function assays were conducted both in vitro and in vivo along with the rescue experiment, for revealing the relative genes' functions in HCC progress. The ChIP and the dual‐luciferase reporter assays were performed to verify the transcriptional regulating profile between E2F7 and SP1/SOX4/Anillin axis. E2F7 was upregulated in HCC and significantly correlated with SP1/SOX4/Anillin axis. High E2F7 expression is associated with dismal clinicopathologic features and poor survival of the patients. E2F7 depletion potently impaired SP1/SOX4/Anillin expression and significantly inhibited HCC growth. Furthermore, intensive exploration demonstrated that E2F7 preserves high SP1 levels by abrogating miR‐383‐5p in a transcriptional way. Atypical E2F7 is an important repressive transcription factor commonly upregulated in the HCC environment. E2F7 facilitates HCC growth by repressing miR‐383‐5p transcription and sequentially promoting SP1/SOX4/Anillin axis. Our findings provide us with probable targets for HCC prevention and therapeutic treatment.

## 1 INTRODUCTION

Worldwide, hepatocellular carcinoma (HCC) ranks third in tumor‐related mortality and gives out researchers and clinical doctors a great challenge.^1^, ^2^ Even though, the recent impressive progress in treating HCC has been achieved through novel strategies, like targeted therapy and tumor immunotherapy, the overall survival (OS) of HCC patients is dismal because of the reckless tumor growth and brutal HCC invasion and metastasis intra‐ or extrahepatic.^3^, ^4^ Thus, it is important and urgent to establish more effective preventing approaches and to discover more precise therapeutic targets for HCC patients.

HCC is endowed with prominent invasive ability due to its strong cell proliferation and tumor growth characteristic.^5^ The cell cycle program participates throughout this whole process as a core event consisting, strictly and orderly controlled.^6^ Accumulating evidence reveals that the axis of cyclin‐dependent kinases (CDKs)–Retinoblastoma protein (RB)‐E2F composes the central mechanism operating cell cycle process.^7^, ^8^ E2F family includes eight distinguished member genes, and as ultimate products and effectors of the above axis, these genes encode transcription factors targeting different genes essential for DNA replication or reparation and exert transcriptional modulation either actively or repressively.

Activation of E2Fs has been identified virtually in all human cancers and is commonly accompanied by complex dysfunction of their upstream regulators, like inactivation of RB Transcriptional Corepressor 1 (RB1), overexpression of CDKs, or the invalidation of the CDK inhibitors.^9^, ^10^ Typically, most of the E2Fs exert active transcription function depending on the control of RB in the process of tumorigenesis and progress. Exceptionally, E2F7 and E2F8 were classified as atypical E2F members due to their specifical effects and activity characteristic independent of RB control, and exert repressive regulation of the genes concerning the cell cycle and proliferation.^11^ Although there have been several reports that suggest the promoting effect of the atypical E2Fs on HCC, the strong evidence of this point along with the exact mechanism is not sufficient and controversial.

Actin‐binding protein Anillin is a key regulator of the cell cycle, and in our previous research, Actin‐binding protein Anillin is a key regulator of the cell cycle, and in our previous research, it has been verified as targeted by the transcription factor sex‐determining region Y‐related (SRY)‐Box Transcription Factor 4 (SOX4) in transcription and playing a potent promoting effect on the process of cytokinesis and tumor growth in HCC.^12^ More than this, we preliminarily noticed that SP1 might participate in SOX4 gene activation. Yet, the thorough upstream regulating mechanism of the SP1/SOX4/Anillin axis in HCC has not been illustrated clearly. Considering that both of the atypical E2Fs and SP1/SOX4/Anillin axis are involved in a similar HCC tumor process, we hypothesized some kind of relationship between these cell cycle regulating genes exists. Accordingly, we detected and observed an obvious upregulation of E2F7 in both HCC tumor tissues and cells, positively correlated with SP1, SOX4, and Anillin. Based on this, we further explored the transcriptional regulation between E2F7 and SP1/SOX4/Anillin axis.

Meaningfully, we not only found the expression correlation in the SP1/SOX4/Anillin axis but also discovered a critical posttranscriptional regulation mediated by E2F7, impacting the effect of microRNA‐383‐5p (miR‐383‐5p) on SP1 messenger RNA (mRNA) degradation. We suggest a pivotal HCC promoting pathway of the SP1/SOX4/Anillin axis under the control of miR‐383‐5p, transcriptional modulated by E2F7.

## 2 MATERIALS AND METHODS

### 2.1 Clinical pathological specimens

A collection of 87 paired specimens, including tumor and paracancerous liver tissues, were prepared from the HCC patients who underwent radical resection without any preoperative treatment between 2014 and 2017, at the Department of General Surgery, Ruijin Hospital, Shanghai Jiao Tong University School of Medicine. The corresponding clinicopathologic parameters of the patients were obtained, including gender, age, tumor size, number of lesions, grades, and so on. An informed consent was obtained. The study was approved by the Ethics Committee of Ruijin Hospital, Shanghai Jiaotong University School of Medicine.

### 2.2 Cell culture

We applied three HCC cell lines (Huh7, HepG2, and Hep3B), and used the normal human hepatic cell LO2 as control (Shanghai Institutes for Biological Sciences, Chinese Academy of Science, Shanghai, China). All of the cells were cultured by RPMI 1640, which was supplemented with 10% heat‐inactivated fetal bovine serum (FBS). All cells were incubated at 37°C environment temperature, with 100 μg/ml streptomycin and 100 U/ml penicillin in a humidified medium, at an atmosphere of 5% CO_2_. Specifically, for the transfected cells, a medium mixed with G418 (Santa Cruz Biotechnology Inc.; 400 μg/ml) was used for selection.

### 2.3 Data sets from Gene Expression Omnibus (GEO), the Cancer Genome Altas (TCGA), and CCLE database preparation

GSE45114 data set from GEO (https://www.ncbi.nlm.nih.gov/geo/) database and the liver cancer data set from TCGA (https://tcga-data.nci.nih.gov/tcga/) database were collected. Platforms of the GSE45114 data set is GPL5918 (CapitalBio Human 22k oligonucleotide microarray), and the data of the microarray were preprocessed through R language and normalized by two professional bioinformatics analysts. TCGA database by USCS Refseq Gene Array containing gene profile of 369 HCC tumor samples and 160 paired adjacent noncancerous samples.

Besides the above data sets, the online database of Cancer Cell Line Encyclopedia (CCLE: https://portals.broadinstitute.org) was analyzed to determine the expression level of candidate genes among differential HCC cell lines. Twenty‐four HCC cell lines were included and explored to determine the expression level of E2F7.

### 2.4 Real‐time quantitative polymerase chain reaction (RT‐qPCR) assay, Western blot analysis, and immunohistochemistry (IHC) assay

RNA isolation from either tissues or cells was conducted according to the instruction of the TRIzol reagent (Invitrogen). The first‐strand complementary DNA (cDNA) was synthesized via High‐Capacity cDNA Reverse Transcription Kit (ABI). All the primers were synthesized (Jike Biotech Company) (Supporting Information: Table 1). RT‐qPCR was operated following the TaqMan Gene Expression Assays protocol (ABI).

Antibodies against the candidate proteins (E2Fs, SP1, SOX4, Anillin) were purchased (Abcam). The Western blot analysis and IHC assay have complied with our previously described methods.^13^ The IHC assay was carried out following our previously described methods.^14^ In brief, the paracancerous tissue sections with 4 μm thickness were cut from paraffin‐embedded tissue blocks, deparaffinized and rehydrated, and treated with 0.01 mol/L citrate buffer (pH 6.0) for antigen retrieval. After blocking with goat serum solution for 45 min, the sections were incubated with primary antibodies at 4°C overnight. Antibodies used for IHC included antibodies against Anillin (1:200; Abcam). After washing three times with PBS, sections were incubated with biotin‐labeled secondary immunoglobulin (1:100; DAKO) for 1 h at room temperature. The sections were then stained with diaminobenzidine (DAKO) and were restained with hematoxylin at room temperature. Two experienced pathologists were assigned independently and blindly for detecting Anillin expression through IHC assay. The specimens were separated into two groups according to the staining intensity grade: no to low staining (0–1+) and moderate to high staining (2+–3+).

### 2.5 Plasmid preparation and cell transfection

The pGU6/Neo vectors containing  short hairpin RNA (shRNA) for E2F7 suppression were transfected into cultured Hep3B cells at the exponential phase (JIKE Biochemistry). The control ones were set up at the same time. All the transfected cells were selected by using a medium mixed with G418 (Santa Cruz Biotechnology Inc.; 400 μg/ml). Similar to this, we also regulated the expression of SP1 in Hep3B cells. The mimic transfection method was used for upregulating miR‐383‐5p ectopically in Hep3B cells (Hep3B/miR‐383‐5p), and the negative control (NigmiR) was set. As for the rescue experiment, miR‐383‐5p mimics were also applied for detecting the influence of E2F7 on SP1 expression and the consequential gene expression changes.

### 2.6 Cell proliferation assay and cell cycle analysis

The treated HCC cells (1 × 10^6^) were cultured in 96‐well microtiter plates triplicated and incubated at an atmosphere of 5% CO_2_ and 37°C for 5 days. Microplate computer software (Bio‐Rad Laboratories Inc.) was applied for measuring the optical density following the Cell Counting Kit‐8 (CCK‐8) assay kit protocol (Dojindo). Then, we plotted the cell proliferation curves. Meanwhile, the cells were treated with ethanol fixation, followed by RNase A treatment and propidium iodide staining. Flow cytometry detection was carried out using FACSCalibur (Becton‐Dickinson) for quantifying cell populations at the G0/G1, S, and G2/M phases, and ModFit software e(Becton‐Dickinson) was used. The debris and fixation artifacts of the cells were excluded.

### 2.7 Cell apoptosis analysis

Cell apoptosis rate was calculated by using PE‐Annexin V Apoptosis Detection Kit I (BD Pharmingen) following the instructions. Transfected cells were resuspended in the concentration of 1 × 10^6^ cells/ml by the 1 × Binding Buffer. Five microlitres of FITC and 5 μl of PI were added to 100 μl of the cell suspension, followed by a 15 min incubation in darkness, with 400 μl × Binding Buffer. The apoptosis rate was calculated through flow cytometry (Becton Dickinson). Both Annexin V‐FITC‐positive and PI‐negative cells were considered apoptosis cells.

### 2.8 Orthotopic transplantation of mouse liver

The 4–5‐week‐old BALB/c nude male mice (Institute of Zoology Chinese Academy of Sciences) were housed in a pathogen‐free environment. Experiment on animal models was performed following the guidelines of the Shanghai Medical Experimental Animal Care Commission. Transfected HCC cells were suspended in 25 µl serum‐free Dulbecoo's modified eagle medium (DMEM) mixed with 25 µl Matrigel (1:1, v/v) every 1 × 10^6^ cell, for orthotopically injecting into the mice's hepatic lobes. All mice were killed 6 weeks after injection, and the xenografted livers were weighted. The livers and lungs from mice were collected for hematoxylin and eosin (HE) independently and detection was conducted by two experienced pathologists.

### 2.9 Chromatin immunoprecipitation (ChIP) assay

ChIP assay was conducted to verify the interaction between the transcription factor and the targeted genes' promoter region. In this study, a total of 5 × 10^6^ cells were cultured in each 10 cm dish and subjected to the protocol of ChIP by using ChIP‐ITTM Kit (Active Motif). Chromatin was immunoprecipitated with 2 μg of either the transcription factor antibodies (Abcam) or IgG as the negative control. The extracted DNA followed was then analyzed through PCR and RT‐qPCR by introducing the relative primers (Supporting Information: Table 1) for amplification of the fragment including the promoter sequences of the targeted genes.

### 2.10 Dual‐luciferase reporter assay

MiR‐383‐5p was suggesetd as an upstream regulator of SP1 according to the prediction result through the online tool of microcosm (http://mirecords.biolead.org). A 202 bp sequence containing the putative binding site of miR‐383‐5p was selected from the 3′‐UTR of SP1 mRNA, along with the mutative sequence (Supporting Information: Table 2). The sequences were respectively cloned into the pMIR‐Report luciferase vector, which contains firefly luciferase, and the pRL‐TK vector luciferase was set as control (Promega). These two sets of vectors were cotransfected into Hep3B cells introducing miR‐383‐5p or the control ones. The luciferase activity was measured via the Dual‐Glo Luciferase assay system (Promega) 48 h after the transfection.

### 2.11 Statistical analysis

Statistical analysis was carried out by using SPSS 20.0. *p*‐values were calculated using an unpaired Student's *t*‐test and Fisher's exact test. Differences were considered statistically significant at *p* < 0.05.

## 3 RESULTS

### 3.1 Ascent of E2F7 was observed in HCC tissues and cells

First of all, we explored the expression profile of E2F7 specifically in HCC tissues compared with the normal tissues throughout the analysis GSE45114 data set from GEO database and the liver cancer data set from TCGA database. We observed an obvious ascent of E2F7 in tissues and the generated heatmap indicated the high expression of E2F7 in tumors (Supporting Information: Figure 1A,B). Similarly, by exploring Cancer Cell Line Encyclopedia (CCLE) database, we also found a general upregulation of E2F7 in most of the HCC cell lines (Supporting Information: Figure 1C).

**Figure 1:**
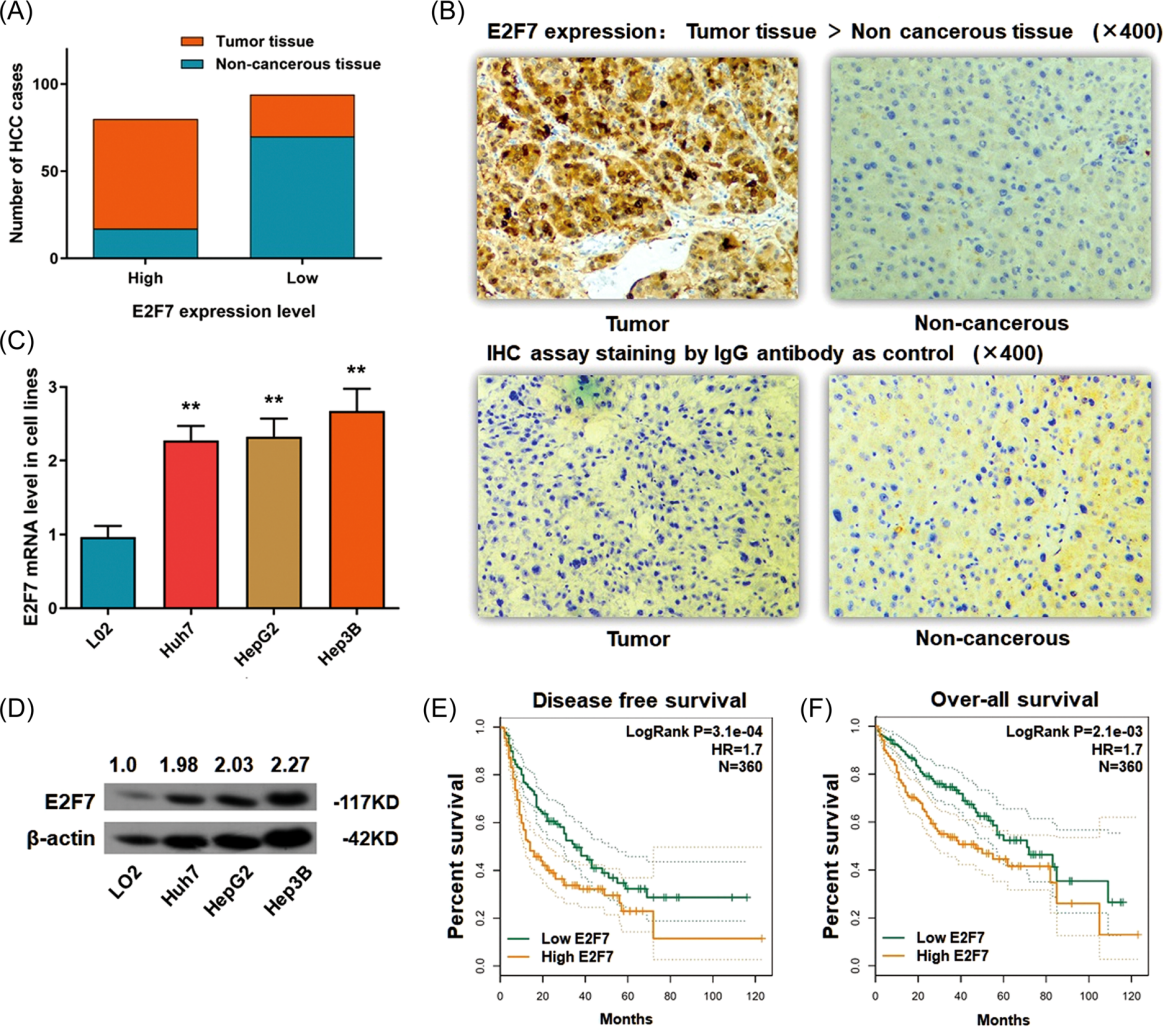
E2F7 ascends in HCC tumor tissues and cell lines. (A) Statistic of the number of cases with higher or lower expression of E2F7 in HCC specimens. E2F7 ascended in most of the tumor tissues (63/87) and was expressed at a lower level in most of the adjacent noncancerous tissues (17/87) (*p* < 0.01). (B) Representative graph of immunohistochemistry analysis (400×) of the HCC cases. The IgG antibody was used for staining the specimens as a control. E2F7 expression in tumor specimens was significantly higher than in adjacent noncancerous tissues. (C) RT‐qPCR assay demonstrated a significantly high expression of E2F7 mRNA in three HCC cell lines, in comparison with the control L02 cells (**p < 0.01). (D) The Western blot analysis demonstrated a remarkable raising of E2F7 protein in three HCC cell lines, in comparison with the control L02 cells (*p* < 0.01). (E) Disease‐free survival (DFS) analysis according to HCC patients' follow‐up information was presented by the Kaplan–Meier plot. High Anillin expression in HCC tissue is correlated with poor overall survival (OS) (360 cases, *p* = 3.1e−04). (F) OS analysis according to HCC patients' follow‐up information was presented by the Kaplan–Meier plot. High Anillin expression in HCC tissue is correlated with poor OS (360 cases, *p* = 2.1e−05). HCC, hepatocellular carcinoma; mRNA, messenger RNA; RT‐qPCR, real‐time quantitative polymerase chain reaction. [Color figure can be viewed at wileyonlinelibrary.com]

On basis of this, we investigated the E2F7 expression status in 87 paired HCC tissues and the adjacent liver tissues from the real patients through the IHC assay and divided the cases into two groups on basis of the expressing intensity of E2F7, namely High E2F7 expression group and Low E2F7 expression group. For tumor tissues, we observed that 72.4% (63/87) of the cases showed E2F7 expression and the rest 27.6% (24/87) ones with a relatively lower Anillin expression. On the contrary, in the adjacent noncancerous tissues, 80.5% (70/87) of the tissues presented a relatively lower expression level of E2F7, and 19.5% (17/87) of the others expressed a higher level of E2F7, which only counted a small portion of the patients (Figure 1A,B).

Consistent with the above findings, the ascent of E2F7 was found in all the three HCC cell lines (Huh7, HepG2, and Hep3B) at both mRNA and protein stages, in comparison with LO2 cells as the control (Figure 1C,D).

The follow‐up information of 361 HCC patients from the TCGA database was carefully reviewed for a thorough understanding of the disease‐free survival (DFS) and the overall survival (OS). Patients with a relatively higher E2F7 level are significantly correlated with dismal DFS rate (log‐rank *p* = 3.1e−04, HR = 1.7) and OS rate (log‐rank *p* = 2.1e−03, HR = 1.7) (Figure 1E,F).

### 3.2 Ascent of E2F7 in HCC correlates with dismal clinicopathological features and poor clinical outcome

The correlations between E2F7 and the clinicopathological features of the 87 HCC patients were calculated. As Table 1 demonstrates, no significant correlation between E2F7 and the patients' age, gender, venous invasion, tumor encapsulation, or hepatitis virus control status was observed. Whereas, the ascent of E2F7 in tumor tissues was significantly correlated with the parameters associated with dismal prognosis and outcome, such as larger tumor diameters, advanced tumor node metastasis classification  stages, tumor microsatellite, venous invasion, and severe liver cirrhosis.

**Table 1:** Correlation between E2F7 transcript and clinicopathological features in 87 HCC specimens

Clinicopathologic parameters	FOXC1 expression		*p* *
	Low (*n* = 24)	High (*n* = 63)	
Age (years)			
≤50	14	32	0.633
>50	10	31	
Gender			
Male	16	32	0.231
Female	8	31	
Diameter (cm)			
≤5	18	25	0.023
>5	9	38	
TNM stage			
I–II	15	17	0.016
III–IV	12	46	
Tumor encapsulation			
Absent	10	26	0.815
Present	17	37	
Tumor microsatellite formation			
Absent	16	21	0.035
Present	11	42	
Venous invasion			
No	14	18	0.054
Yes	13	45	
HBsAg			
Negative	5	6	0.295
Positive	22	57	
AFP (ng/ml)			
≤400	17	3	<0.001
>400	10	60	
Cirrhosis			
Absent	5	5	0.159
Present	22	58	

*Note*: E2F7 level associated with clinicopathologic features in 87 HCC patients, including age, gender, tumor size, tumor stage (AJCC), tumor encapsulation, tumor microsatellite formation, vein invasion, HBsAg status, AFP level, and liver cirrhosis. Statistical significance was assessed by Fisher's exact text.

Abbreviation: AFP, alpha‐fetoprotein; HCC, hepatocellular carcinoma.

* *p* < 0.05.

### 3.3 Depletion of E2F7 impairs cell proliferation of Hep3B cells, induces cell apoptosis in vitro, and represses tumor growth in vivo

**Figure 2:**
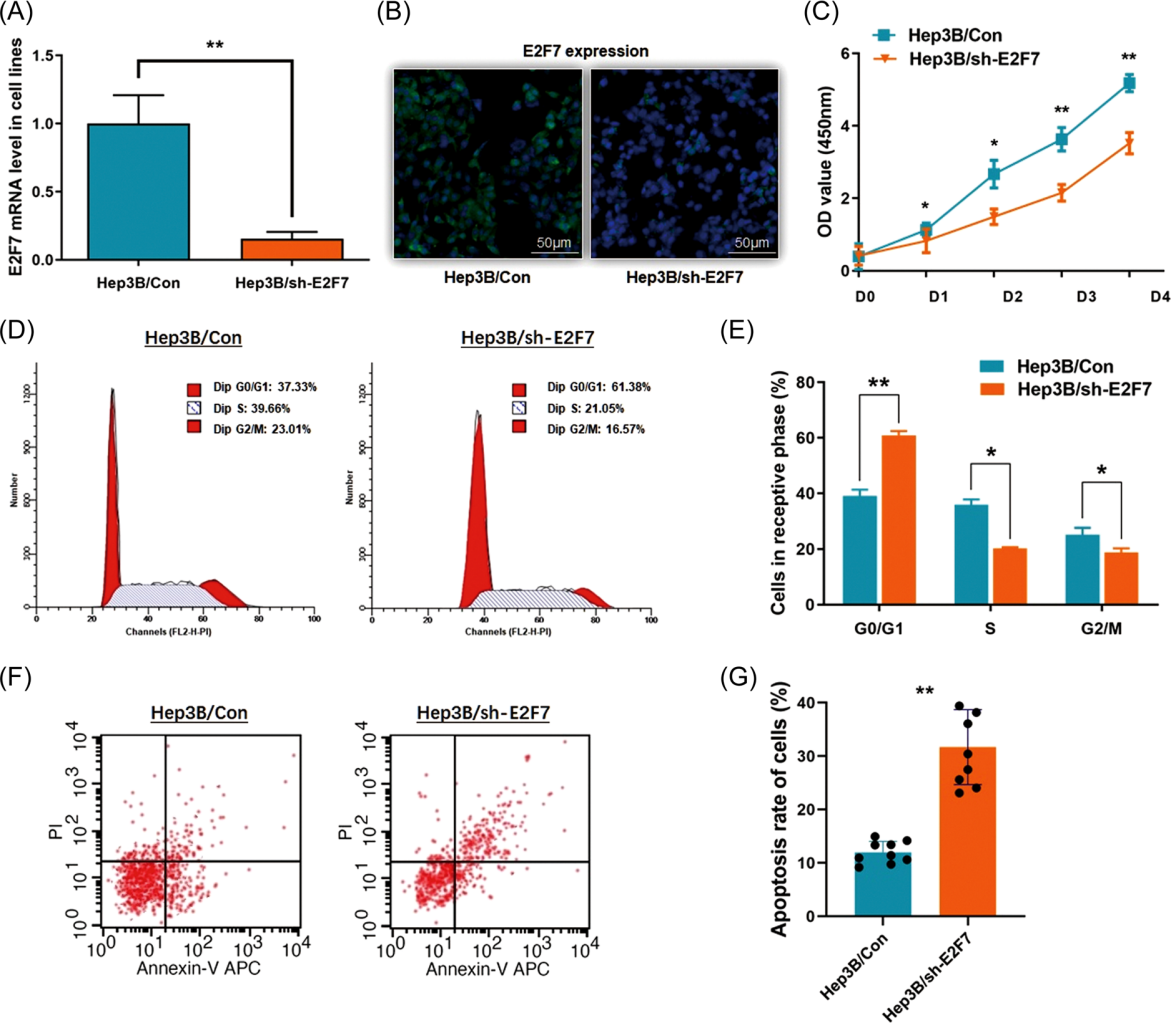
E2F7 depletion impairs cell proliferation and induces cell apoptosis in Hep3B cells. (A) E2F7 was depleted through shRNA transfection in Hep3B cells. RT‐qPCR assay indicated a significant descent of E2F7 mRNA level in the treated cells (**p < 0.01). (B) Validation of the effect of E2F7 depletion through immunofluorescence detection demonstrates a significant decrease of E2F7 protein. (C) The CCK8 assay was applied for investigating the cell proliferation ability. Cell proliferation in Hep3B cells was significantly blocked after E2F7 depletion (*p < 0.05, **p < 0.01). (D) The representative histograms describe the cell cycle profiles of Hep3B cells by using flow cytometry. (E) The cell cycle of Hep3B cells was arrested in G0/G1 phase by depleting E2F7. The results are means of three independent experiments ± SD. (*p < 0.05, **p < 0.01). (F) The representative histograms for describing cell apoptosis profile in Hep3B cells through flow cytometry. (G) The apoptosis rate of Hep3B cells was significantly increased from 11.98% to 31.69% (**p < 0.01), while E2F7 was depleted. The results are means of three independent experiments ± SD. (**p < 0.01). CCK8, Cell Counting Kit‐8; mRNA, messenger RNA; RT‐qPCR, real‐time quantitative polymerase chain reaction. [Color figure can be viewed at wileyonlinelibrary.com]

Hep3B cells presented the highest E2F7 expression among the three HCC cell lines in this study, and we successfully used an shRNA method to deplete E2F7 in this cell line (Figure 2A,B). The loss‐of‐function assay was conducted for observing the cell proliferation, cell cycle changes in vitro, and also the tumor growth capacity in the orthotopic xenograft mouse model.

As we observed from the results when depleting E2F7 in Hep3B cells, the CCK8 assay demonstrated a significant suppression of cell proliferation, of which the *p*‐value was <0.05 for Day 1, and <0.01 for Days 3–4 (Figure 2C). The percentage of Hep3B cells arrested at the G0/G1 phase was increased (from 30.01% to 60.91%; *p* < 0.01). And correspondingly, the cells that went into the S phase cells and the G2/M phase were remarkably decreased respectively from 35.85% to 20.29% (*p* < 0.05) and from 34.14% to 18.80% (*p* < 0.05) (Figure 2D,E).

The flow cytometric analysis was conducted to validate the effect of E2F7 on HCC cell apoptosis. Figure 2E,F shows an effective acceleration of cell apoptosis in Hep3B cells when E2F7 depletes, with the average apoptosis rate raised to 31.69% from 11.98% (*p* < 0.01) (Figure 2F,G).

**Figure 3:**
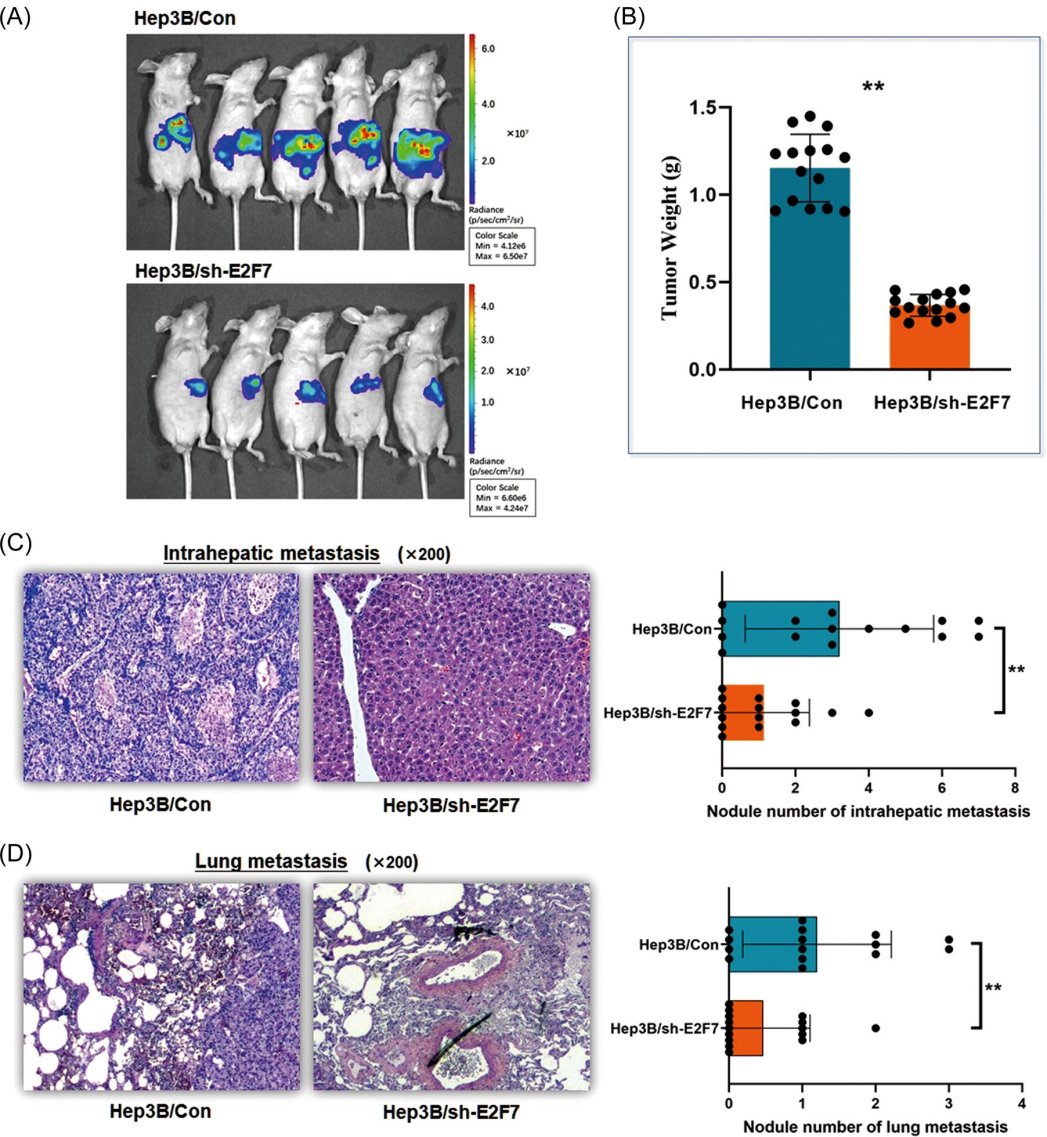
E2F7 depletion in Hep3B cells suppresses tumor invasion and metastasis in an orthotopic xenograft mouse model. Mouse liver orthotopic transplantation model was constructed through 4–5‐week‐old male BALB/c nude mice. (A) The orthotopic tumor growth was significantly suppressed in the mice treated with E2F7 depleted Hep3B cells. (B) Depletion of E2F7 in Hep3B cells led to a significant decrease in the xenograft tumor weight (**p < 0.01). (C) The xenograft tumor specimens were collected and checked under HE staining examination. Depletion of E2F7 in Hep3B cells induced fewer intrahepatic metastasis lesions in the mouse models than in the control ones (**p < 0.01). (D)Depletion of E2F7 in Hep3B cells resulted in fewer metastasis lesions in the lung of the mouse models than in the control (**p < 0.01). HE, hematoxylin and eosin. [Color figure can be viewed at wileyonlinelibrary.com]

Xenograft mouse models were investigated. The quantification of the orthotopically transplanted masses in the liver was measured 6 weeks postorthotopic injection of the transfected Hep3B cells, and the result demonstrated much smaller tumor masses generated in mouse livers when E2F7 was depleted (Figure 3A,B). The HE staining examination indicated that fewer intrahepatic and lung metastasis formations were found in the mice models with lower E2F7 expression than the control ones (Figure 3C,D). The findings above suggested that E2F7 was an effective enhancer of HCC tumor growth, and E2F7 might be a potential breakthrough for HCC‐targeted study.

### 3.4 SP1 activates SOX4 gene transcription in HCC cells

**Figure 4:**
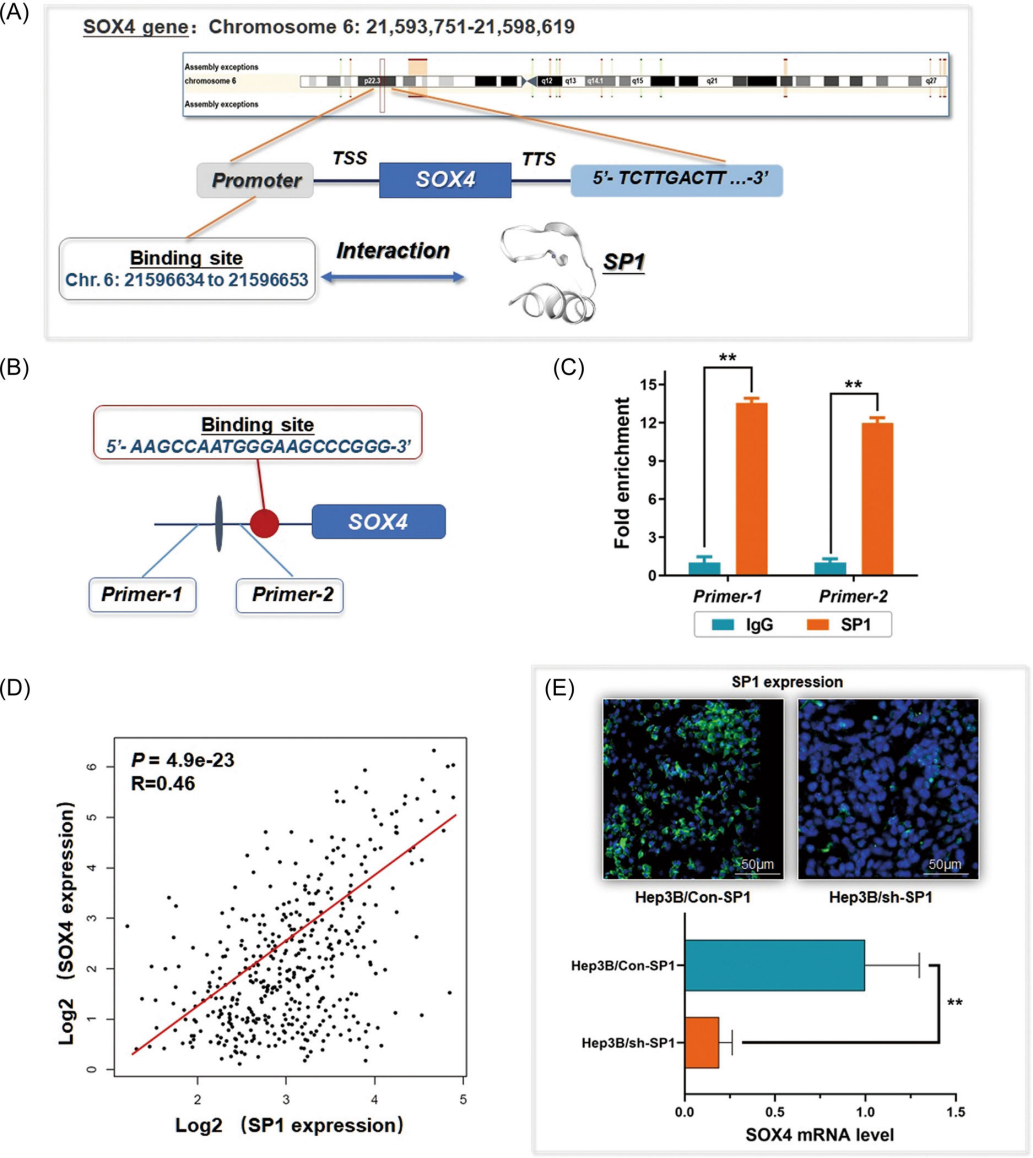
Transcription of SOX4 gene is activated by SP1 in HCC cells. (A) SP1 is a potential transcription factor that binds to the promoter region of the SOX4 gene (5′‐AAGCCAATGGGAAGCCCGGG‐3′, Chr. 6: 21596634 to 21596653). (B) and (C) ChIP assay was carried out for investigating the direct interaction between SOX4 and Anillin gene promoter (**p < 0.01). IgG was used as the negative control. (D) Analysis of the TCGA liver cancer data set demonstrates a significant positive expression correlation between SP1 and SOX4 (*p* = 4.9e−23). (E) Depletion of SP1 in Hep3B cells was validated through immunofluorescence detection and induced a significant descent of SOX4 mRNA level (**p < 0.01). mRNA, messenger RNA; TCGA, the Cancer Genome Altas. [Color figure can be viewed at wileyonlinelibrary.com]

A fragment of 3000 bp from the SOX4 gene promoter sequence was collected for predicting valuable transcription factor binding sites activating the SOX4 gene. By using and screening the results obtained from the Database of Human Transcription Factor Targets (http://bioinfo.life.hust.edu.cn/hTFtarget#!/) and Gene‐Cloud of Biotechnology Information (GCBI; https://www.gcbi.com.cn), we noticed SP1 as the transcription factor of SOX4, binding to the gene promoter region at a specific sequence (5′‐AAGCCAATGGGAAGCCCGGG‐3′, Chr. 6: 21596634 to 21596653) with significance (*p* = 1.29e−07) (Figure 4A). The definite interaction between SP1 and SOX4 gene was validated through ChIP assay as shown in Figure 4B,C. A positive correlation between SP1 and SOX4 was observed through TCGA liver cancer data (*n* = 361, *p* = 4.9e−23, *R* = 0.46) (Figure 4D). Simultaneously, a significant decrease of SOX4 mRNA was found in the Hep3B cells with SP1 knockdown through the shRNA method (Figure 4E). Thus, we concluded that SP1 played the role of the upstream regulator of SOX4, and composed an axis of SP1/SOX4/Anillin in promoting HCC.

### 3.5 MicroRNA‐383‐5p negatively regulates SP1 mRNA level and is targeted by E2F7

**Figure 5:**
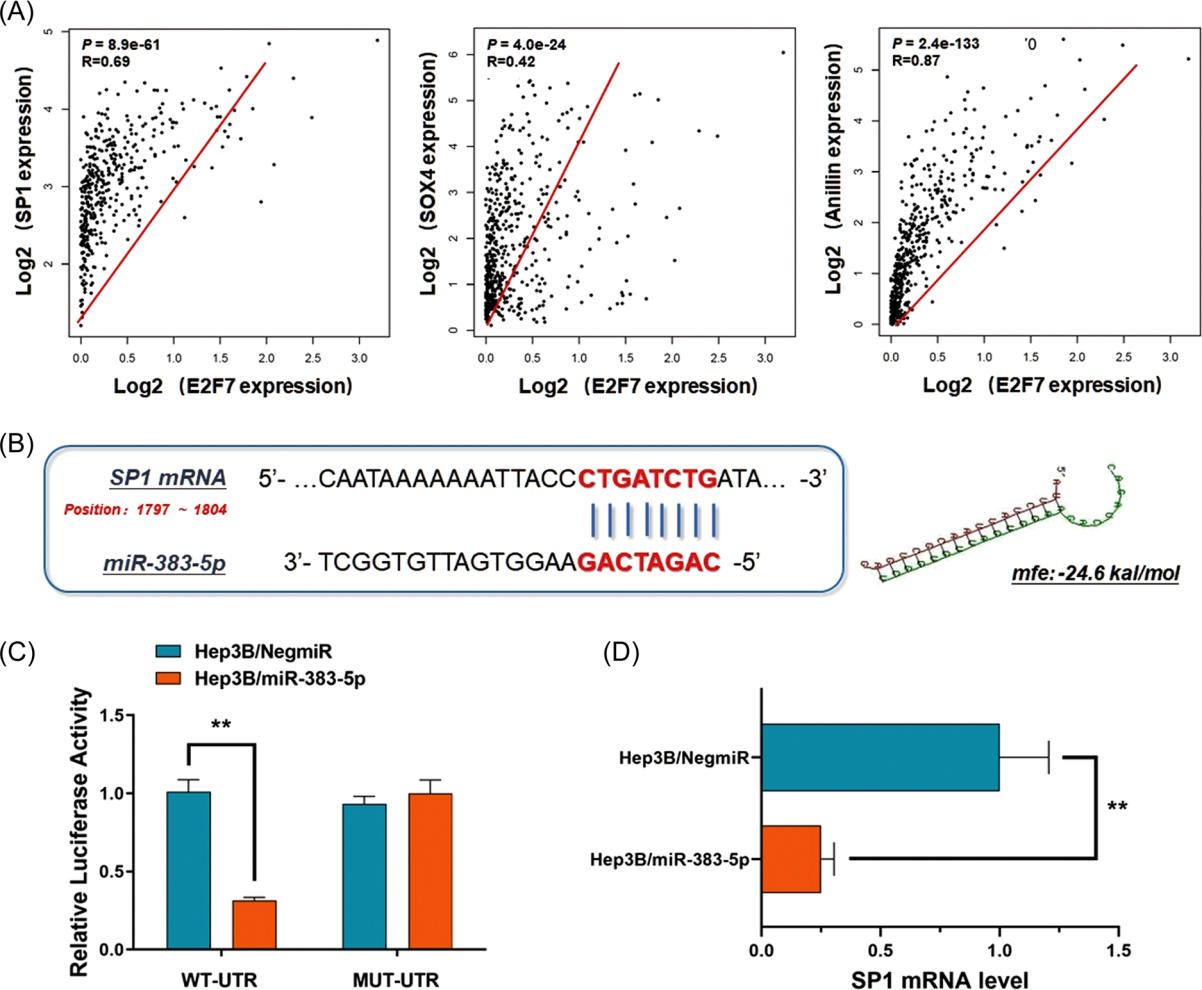
MicroRNA‐383‐5p negatively regulates SP1 mRNA level in a posttranscriptional way, and potentially correlated with E2F7. (A) According to the analysis of the TCGA liver cancer data set (*n* = 361), E2F7 was highly expressed in HCC cells, and presented a positive correlation with SP1 (*p* = 8.9e−61, *R* = 0.69), SOX4 (*P* = 4.0e−24, *R* = 0.42), and Anillin (*p* = 2.4e−133, *R* = 0.87), prompting some regulation mechanisms between E2F7 and SP1/SOX4/Anillin axis. (B) MiR‐383‐5p was a potential upstream regulator of SP1 in Hep3B cells by directly interacting with the 3′‐UTR of SP1 mRNA. The minimum free energy (Mfe) hybridization is calculated with significance as −24.6 kal/mol. (C) A direct interaction was detected between SP1 mRNA and miR‐383‐5p via a Dual‐luciferase reporter assay. Ectopic expression of miR‐383‐5p in Hep3B cells (Hep3B/miR‐383‐5p) significantly declined the luciferase signal of wildtype binding sequence compared with the negative control (Hep3B/NigmiR). The signal suppression induced by miR‐383‐5p defected in Hep3B cells transfected with the mutated binding sequence (**p < 0.01). (D) RT‐qPCR assay indicated a significant decrease of SP1 mRNA level in Hep3B cells, transfected with miR‐383‐5p mimics (**p < 0.01). HCC, hepatocellular carcinoma; mRNA, messenger RNA; RT‐qPCR, real‐time quantitative polymerase chain reaction; TCGA, the Cancer Genome Altas. [Color figure can be viewed at wileyonlinelibrary.com]

As we observed, E2F7 was highly expressed in HCC cells and presented a positive correlation with SP1, SOX4, and Anillin (Figure 5A). Thus, we wondered if there exist some regulation mechanisms between E2F7 and SP1/SOX4/Anillin axis. MiR‐383‐5p has been verified to be decreased in HCC tissues and was suggested as an HCC suppressive modulator in our previous research. By exploring the potential downstream targets, we noticed several highly differentially expressed genes in HCC, including AKR1B10 and SP1 (Figure 5B, Supporting Information: Figure 2). In our latest study, we verified AKR1B10 as an oncogene and targeted by miR‐383‐5p in HCC.^14^ However, SP1 mRNA needed to be further validated.

In this study, Hep3B cells transfected with miR‐383‐5p mimics were prepared. Meanwhile, the vectors containing the fragment of SP1 mRNA 3′‐UTR (WT‐UTR) and the control luciferase vectors containing the mutated sequence (MUT‐UTR) were established. The Dual‐luciferase reporter assay was carried out, and ectopic expression of miR‐383‐5p (Hep3B/miR‐383‐5p) significantly declined the luciferase signal of SP1/pMIR/WT in Hep3B cells compared with the negative control (Hep3B/NigmiR). The signal suppression induced by miR‐383‐5p defected in Hep3B cells transfected with mutated binding sequence, along with a sequential decrease of SP1 mRNA expression by upregulating miR‐383‐5p (Figure 5C,D). The results above supported the direct binding and degradation effect of miR‐383‐5p on SP1 mRNA.

**Figure 6:**
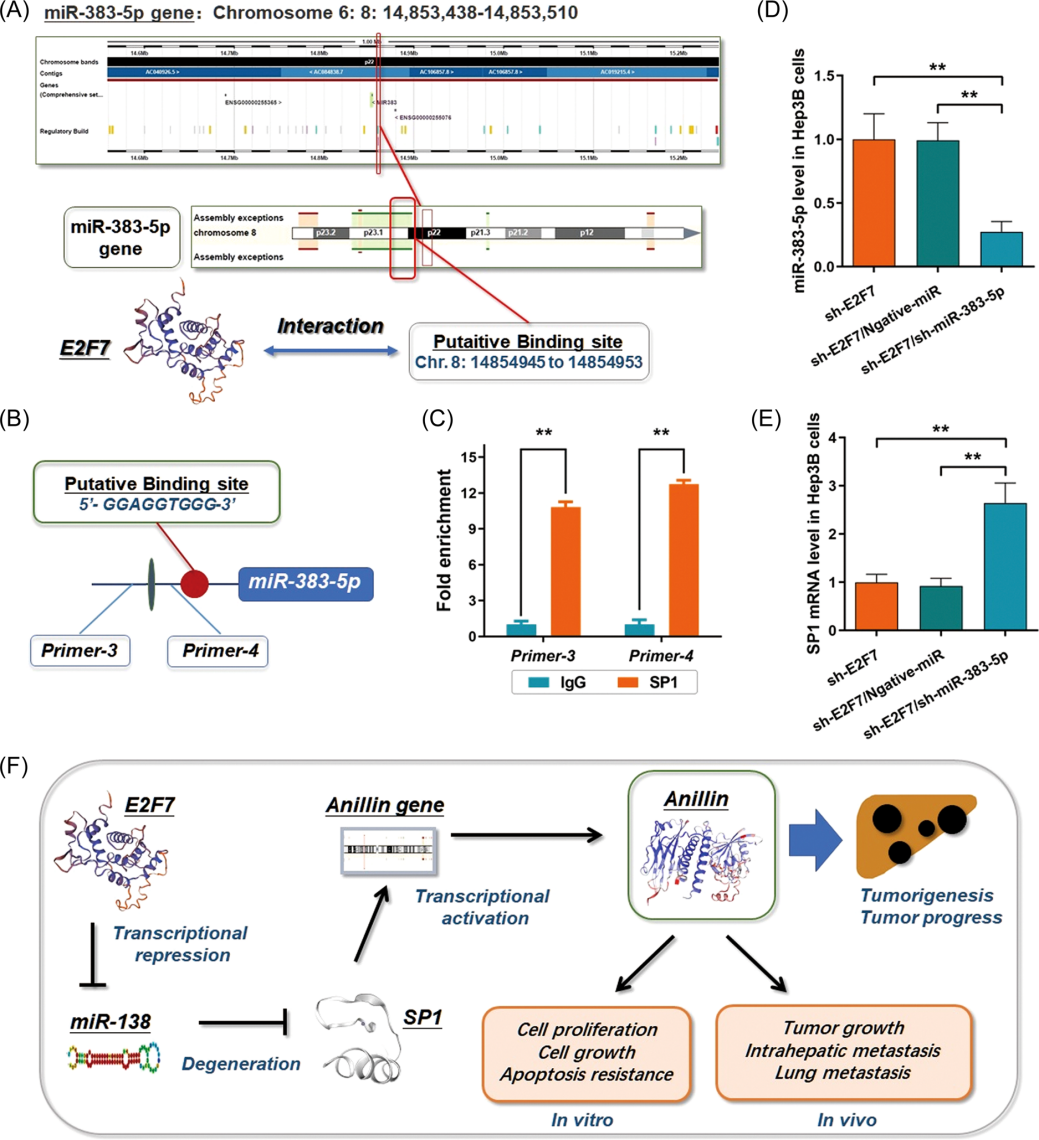
E2F7 transcriptionally targets miR‐383‐5p and modulates the SP1/SOX4/Anillin axis. (A) E2F7 was predicted as a transcription factor that binds to the upstream miR‐383‐5p gene (5′‐GGAGGTGGG‐3′, Chr. 8: 14854945 to 14854953, *P* = 2.89e‐05). (B) and (C) ChIP assay was carried out for investigating the direct interaction between E2F7 and the putative promoter region upstream miR‐383‐5p gene (**p < 0.01). IgG was used for the negative control. (D) MiR‐383‐5p presented a relatively higher level in the E2F7 depleted Hep3B cells. And this high expression of miR‐383‐5p was significantly through the shRNA method (**p < 0.01). (E) By suppressing miR‐383‐5p, the downregulation of SP1 mRNA induced by E2F7 depletion was significantly rescued (**p < 0.01). (F) Summary of the regulation of E277 on the axis of SP1/SOX4/Anillin by negatively modulating the transcription of miR‐383‐5p, which sequentially facilitates HCC tumor growth. HCC, hepatocellular carcinoma; ChIP, chromatin immunoprecipitation; mRNA, messenger RNA. [Color figure can be viewed at wileyonlinelibrary.com]

Intriguingly, in the Hep3B cells with E2F7 depletion, miR‐383‐5p was sequentially upregulated. Considering E2F7 is an effective repressive transcription factor, we applied similar methods to the previous section to learn the transcriptional regulation of miR‐383‐5p. As expected, we discovered a potential binding site upstream of the sequence of the miR‐383‐5p gene specifically interacting with E2F7 (5′‐GGAGGTGGG‐3′, Chr. 8: 14854945 to 14854953, *p* = 2.89e−05) (Figure 6A,B). To give more evidence of this repressive transcriptional regulation, the direct interaction between E2F7 and the upstream sequence of miR‐383‐5p was validated according to the results generated from the ChIP assay (Figure 6C). Moreover, we applied a small interfering RNA (siRNA) against miR‐383‐5p to conduct the rescue experiment in Hep3B cells with E2F7 depletion. As Figure 6D,E shows, by suppressing miR‐383‐5p, the downregulation of SP1 mRNA induced by E2F7 depletion was significantly rescued.

Taken together, we suggested that E2F7 exerted the effect of preserving the SP1/SOX4/Anillin axis to facilitate HCC growth through a repressive transcriptional modulation on its target miR‐383‐5p. The brief pattern diagram is presented in Figure 6F.

## 4 DISCUSSION

The tough aggressiveness of HCC, including tumor cell growth, invasion, and metastasis leads to unsatisfactory prognosis and miserable outcomes for the patients, even if radical operations have been conducted.^15^ So it is an important issue to learn much more key points and core mechanisms of the strong cell proliferation ability of HCC tumor cells to seek potential targets and treatments therapeutically.^16^

Our previous research had discovered a solidated regulation between SOX4 and Anillin in HCC. Anillin is an evolutionarily conserved regulator, which exerts critical functions on promoting cytokinesis to generate two daughter cells at the end stage of mitosis. In different cancers like lung, breast, and ovarian, SOX4 has been described as an oncogene involves in multiple tumorigenesis and progress pathways, overlaps with Wnt, PI3K, and TGF‐β signaling.^17^, ^18^, ^19^ Consistent with these findings, we observed that a remarkable upregulation of Anillin in HCC, and the xenograft experiment with Anillin depletion in HCC cells indicated an effective abrogation of tumor growth ability in situ, consistent with an obvious depression of HCC cell proliferation and the arrest of cell cycles.

On basis of this, we proposed Anillin as an effector, which could be probably targeted for HCC treatment. Meanwhile, we observed a significant upregulation of SOX4 at both mRNA and protein stages. As acknowledged, SOX4 is a member of the SRY high‐mobility group (HMG) box family and the group C subclass, which plays the role of essential transcription factor associated with the modulation of cell stemness, differentiation, and progenitor development.^20^, ^21^ The specific HMG box domain endows SOX4 an effective binding ability to the groove of its downstream genes and consequentially modulates these genes' transcription activities.^22^ In different cancers like lung, breast, and ovarian, SOX4 has been described as an oncogene involves in multiple tumorigenesis and progress pathways, overlaps with Wnt, PI3K, and TGF‐β signaling.^23^, ^24^ Interestingly, in our previous study, the Anillin mRNA level showed a swift change when we depleted SOX4 in HCC cells, and the tumor growth was impaired as a result. Considering SOX4's transcriptional functions, we conducted the ChIP assay, by which the direct interaction between SOX4 and the Anillin gene promoter region was validated. We suppose that SOX4 and Anillin play positive roles in HCC tumor growth synergistically. Since there had been no definite regulation upstream SOX4/Anillin reported before, we believe that learning the related regulating mechanism or singling pathway can provide valuable theoretical support for developing new targeting therapy strategies.

SP1 has been described as a promising oncogene in multiple human malignancies, which exerts strong promoting effects on cancer growth, metastasis, and even chemoresistance.^25^, ^26^ For instance, in ovarian cancer, SP1 promotes LncRNA‐DANCR‐induced tumor cell proliferation through a direct transcriptional activation.^27^ In colon cancer, SP1 induces drug resistance to 5‐Fu via modulating the members of the Mitogen‐actived protein kinase (MAPK) signaling pathway.^28^ There is also some evidence supporting the tumor improvement caused by SP1 in HCC. As an example, SP1 is the upstream transcription factor of miR‐130b, which promotes HCC angiogenesis and tumor growth by degrading tumor suppressor HOXA5.^29^ Similarly, SP1 leads to RasGRP1 upregulation in HCC cells and sequentially induces the enhancement of cell proliferation.^30^ Whereas, among the complex regulation events, there was no sufficient evidence reported between SP1 and SOX4 or Anillin. In this study, the application of online prediction software provided a specifical binding site of SP1 at the promoter region of the SOX4 gene with significant value, and we validated the exact transcriptional interaction resulting in an effective activation of the SOX4/Anillin axis in HCC tumor growth. Combined with the series of our research, we suggest an axis composed of SP1/SOX4/Anillin as another novel pathway promoting HCC growth.

As mentioned ahead, the members of the E2F family are complex in the composition of the various genes with numerous isoforms.^8^ Generally, they could be tendentiously grouped according to the effects on transcription as the canonical activators (E2F1, E2F2, and E2F3a), the canonical repressors (E2F3b, and E2F4–E2F6), and the atypical repressor including E2F7 and E2F8.^31^ Although there has been definite evidence to prove the importance of E2Fs in cell development, the overview and comprehensive understanding of this family in physiology or disease process is fragmented and remains tremendously uncertain, especially for the atypical members, E2F7 and E2F8.

In this study, according to the expression correlation and functional experiments, we focused on E2F7 and supposed that some probable pathways exist in HCC progress concerning E2F7 and the SP1/SOX4/Anillin axis. E2F7 is involved in the cell cycle mainly playing a repressive transcriptional function. Whereas, the effects of E2F7 on human cancers are controversial because of its ambiguous functions ever reported. For instance, E2F7 binds the putative promoter region of the miR‐199b gene and significantly repressed the expression of this tumor‐suppressing miRNA in colon cancer, which leads to the increase in its target USP47, and sequentially enhances the MAPK signaling pathway and facilitates tumor progress.^32^ Similar to this, E2F7 exerts an actively transcriptional effect, but not repressively as general, was found in pancreatic cancer, and promotes tumor cell proliferation by efficient enhancement of VEGFa transcription.^33^

Oscillatory effects of E2F7 on HCC were also reported. Recently, Moreno et al.^34^ proposed that E2F7 inhibited HCC tumor growth in adult mice by overriding cell‐cycle entry and against the effects of the other E2F members. However, on the contrary, other researchers gave out the opposite opinion. It was reported that E2F7 transcriptionally regulated the VEGFR2 signaling pathway and induced HCC angiogenesis and tumor proliferation.^35^ Similarly, overexpressed E2F7 could significantly impact AKT/β‐catenin/CCND1 signaling pathway and promote the liver cancer stem cells to self‐renew and cell cycle entry.^36^ Taken together, we suppose that E2F7 may mediate different tumor‐related effects or events through its transcriptional function in a target‐dependent way, and the overall effect of E2F7 on HCC progress should be discussed comprehensively, and the accumulated evidence of different pathways or regulation axis may help us to learn more about this gene.

In this study, we observed that E2F7 was highly expressed in both HCC tumor tissues and cells, and presented an inverted correlation with the PFS and OS of the patients. Highly expressed E2F7 pointed to dismal clinicopathologic features. And depletion of E2F7 led to significant impairment of cell proliferation and tumor growth. Considering these findings, it could be suggested that E2F7 plays much like an enhancer rather than a suppressor in HCC.

Noteworthily, we also found that E2F7 appeared to be positively related to SP1/SOX4/Anillin axis at the expression level. And miR‐383‐5p, a posttranscriptional regulator discovered in our previous research as a tumor suppressor in HCC, was predicted as a potential bridge that mediates the regulation of E2F7 on SP1. And we propose this hypothesis as the key point for illustrating the function of E2F7 in this study.

As acknowledged, miR‐383‐5p plays a critical role as a suppressor in a variety of human malignancies, including lung cancer, gastric cancer, and colorectal cancer, by degrading different mRNAs of the oncogenes, such as EPAS1, ERBB4, and CREB1.^37^, ^38^, ^39^ For HCC, the related literature is limited. One piece of work in our study has demonstrated that miR‐383‐5p suppresses tumor growth through directly binding to and degrading AKR1B10 mRNA.^14^ And recently, another study proposed that miR‐383‐5p attenuates the chemoresistance of oxaliplatin in HCC by degrading VAMP2, and also suppresses tumor growth.^40^ Based on these findings, we further discovered and validated SP1 mRNA as a downstream target negatively regulated by miR‐383‐5p through direct interaction. And the decrease of miR‐383‐5p should be one of the causes for explaining the preservation and activation of the SP1/SOX4/Anillin axis in HCC progress. Second, the suppressive translational regulation of E2F7 on miR‐383‐5p was verified through the ChIP assay, consistent with the impressive recovery of SP1 expression, when E2F7 was depleted in HCC cells. And thus, the transcriptional modulation on SP1/SOX4/Anillin axis by E2F7 could be formulated.

In summary, as the atypical member of the E2F family, E2F7 facilitates HCC cell proliferation and tumor growth. Among the complex functions in the tumor cell, E2F7 exerts the repressive translational regulation on miR‐383‐5p. And by this way, E2F7 mediated the preservation of SP1 mRNA, which sequentially boosts HCC progress in the manner of the SP1/SOX4/Anillin axis. The findings we described above probably provide us with an innovative and potential strategy for HCC prevention and treatment.

## AUTHOR CONTRIBUTIONS

Nan Wang and Junqing Wang wrote the article. Wen Xu contributed to the data analysis and biomolecular experiments. Xiaochun Fei was in charge of the pathological experiments and data mining. Yongjun Chen worked for clinicopathological features collection. Fengjie Hao and Junqing Wang designed and directed the study.

## CONFLICT OF INTEREST

The authors declare no conflict of interest.

## Supporting information

Supporting information.

## Data Availability

The data that support the findings of this study are available from the corresponding author upon reasonable request.
