# Supplementary material for: E2F7 enhances hepatocellular carcinoma growth by preserving the SP1/SOX4/Anillin axis via repressing miRNA‐383‐5p transcription
Source: Mol Carcinog. 2022 Aug 4;61(11):975–88. doi: 10.1002/mc.23454 (PMC9804269; doi:10.1002/mc.23454)
Supplement: Supplementary file 1 — Supporting information. [file MC-61-975-s001.docx]

**Suppl. Table. 1**

**The primers for RT-qPCR assay**

| **Genes** | **Forward** | **Reverse** |
| --- | --- | --- |
| **E2F7** | **5’-GCAGTGGTTGTTTCTGTCAGG**  **-3’** | **5’-CTGGTCAGTGTAGGGCACA**  **-3’** |
| **SP1** | **5’-CCCTTGAGCTTGTCCCTCAG-3** | **5’-GTAGCCCCAGAGGAGGAAGA**  **-3** |
| **SOX4** | **5’-CCCAGCAAGAAGGCGAGTTA**  **-3’** | **5’-CCTTCCAGTTCGTGTCCTCC-3’** |
| **ChIP assay**  **Primer-1** | **5’-CCGTTCCAAGCCCCTTGTTA-3’** | **5’-GAAACAGGCCTCTTCCCCTC-3’** |
| **ChIP assay**  **Primer-2** | **5’-TGACAGGTGTCTAGGTACCACT-3’** | **5’-CGTGTTCTCGGCATTGTTGG-3’** |
| **ChIP assay**  **Primer-3** | **5’-TGAGAATCGCTTGAGCCCAG-3’** | **5’-AAGTCGGGAGCGGGATTTC-3’** |
| **ChIP assay**  **Primer-4** | **5’-TGGAGATGCCTAGGAAGTTGC-3’** | **5’-AGGAGAGAGTAGGTCAATTGGGA-3’** |
|  |  |  |

**Suppl. Table. 2**

**Selected sequence of the predicted miR-383-5p binding site of the 3’-UTR of SP1 mRNA, along with the relative mutated sequence**

| **Genes** | **Sequence including the binding site**  **(202 bp)** | **Relative mutated sequence** |
| --- | --- | --- |
| **SP1 mRNA**  **3’-UTR** | 5’-tgtgtgtgtgtgtgtgtgtgtgtgtgtgtaatctgttaggttggggataggttttctgctagccaatattaaaagagacctgcaataaaaaaattaccctgatctgatagaaagcaagtgtttttgtatgtgtgggtgaatgtgtgttcatgcccgtatatgtctacacacagatgacaaattatatttgaaatcgttggaa-3’ | 5’-tctctctctctctctctctctctctctcttaacagattgctagcgcaaacgatatgtccaaccgattttaatatgtgtcgtcctaaatatataattcgcagttgtcaaacatacctactctatatctttctctcgctcattctctctacttccgcctttttctgttctctctgttcagataatttttatcataacctagcat-3’ |
|  |  |  |

**Supplementary Figures**

**Suppl. Fig. 1**


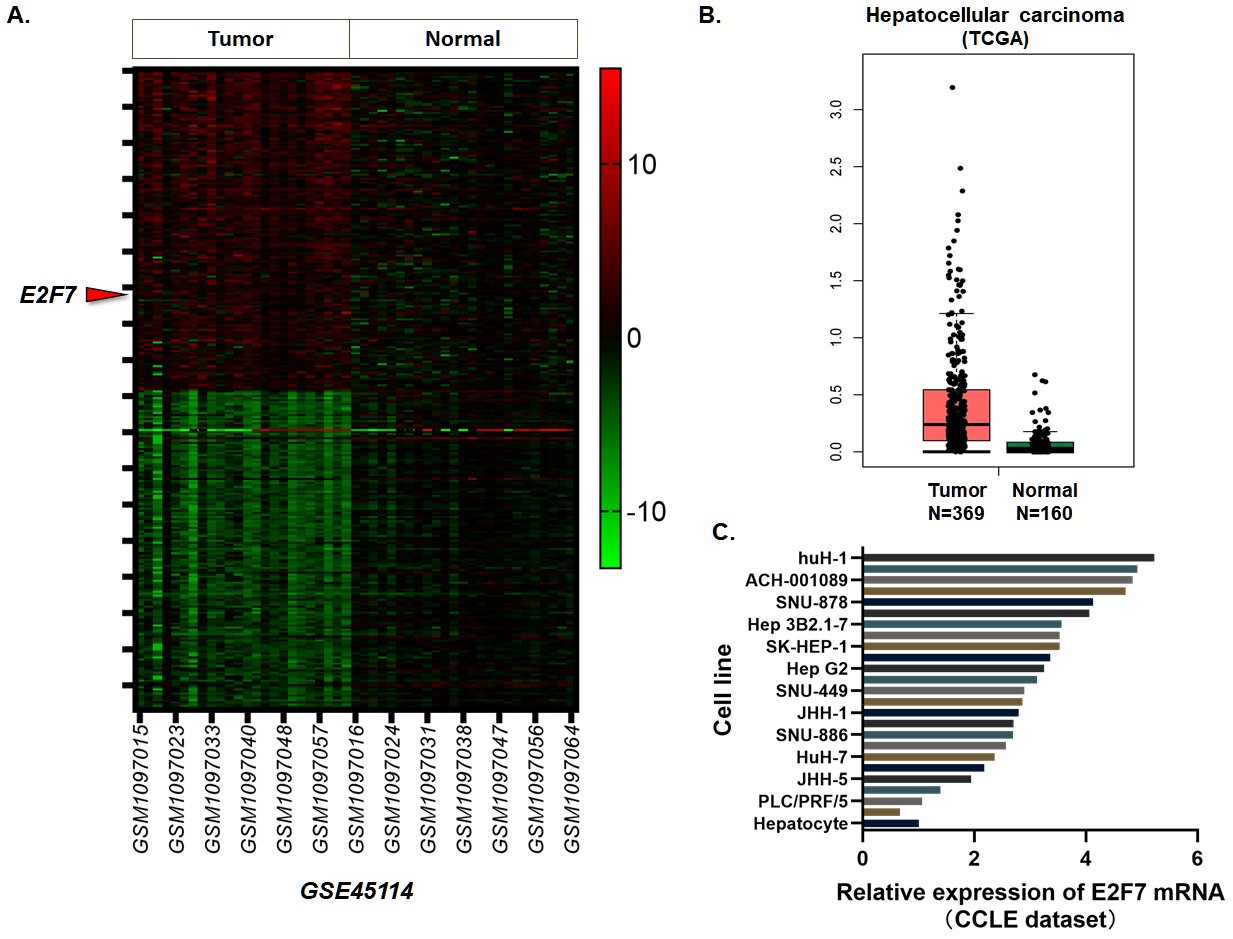


**Suppl. Fig. 1. E2F7 expression profile obtained from the analysis of GEO, TCGA, and CCLE datasets**

**A.** HCC patients’ information and gene expression data from 45 cases were collected from the GEO GSE45114 dataset. Heatmap was generated and listed the top 150 differentiation expression genes either up-regulated or down-regulated in HCC, E2F7 was included and presented a remarkably high expression in tumor tissues compared with the normal liver tissue (*P*=1.5e-03). B. The gene expression data of HCC patients were collected from TCGA liver cancer datasets including 369 HCC and 160 normal liver tissue specimens’ information. E2F7 was significantly up-regulated in HCC tissues (*P*＜0.001). C. The expression levels of E2F7 were detected in multiple HCC cell lines from the CCLE database. E2F7 is significantly up-regulation of E2F7 in most of the HCC cell lines compared with the normal hepatocytes.

**Suppl. Fig. 2**


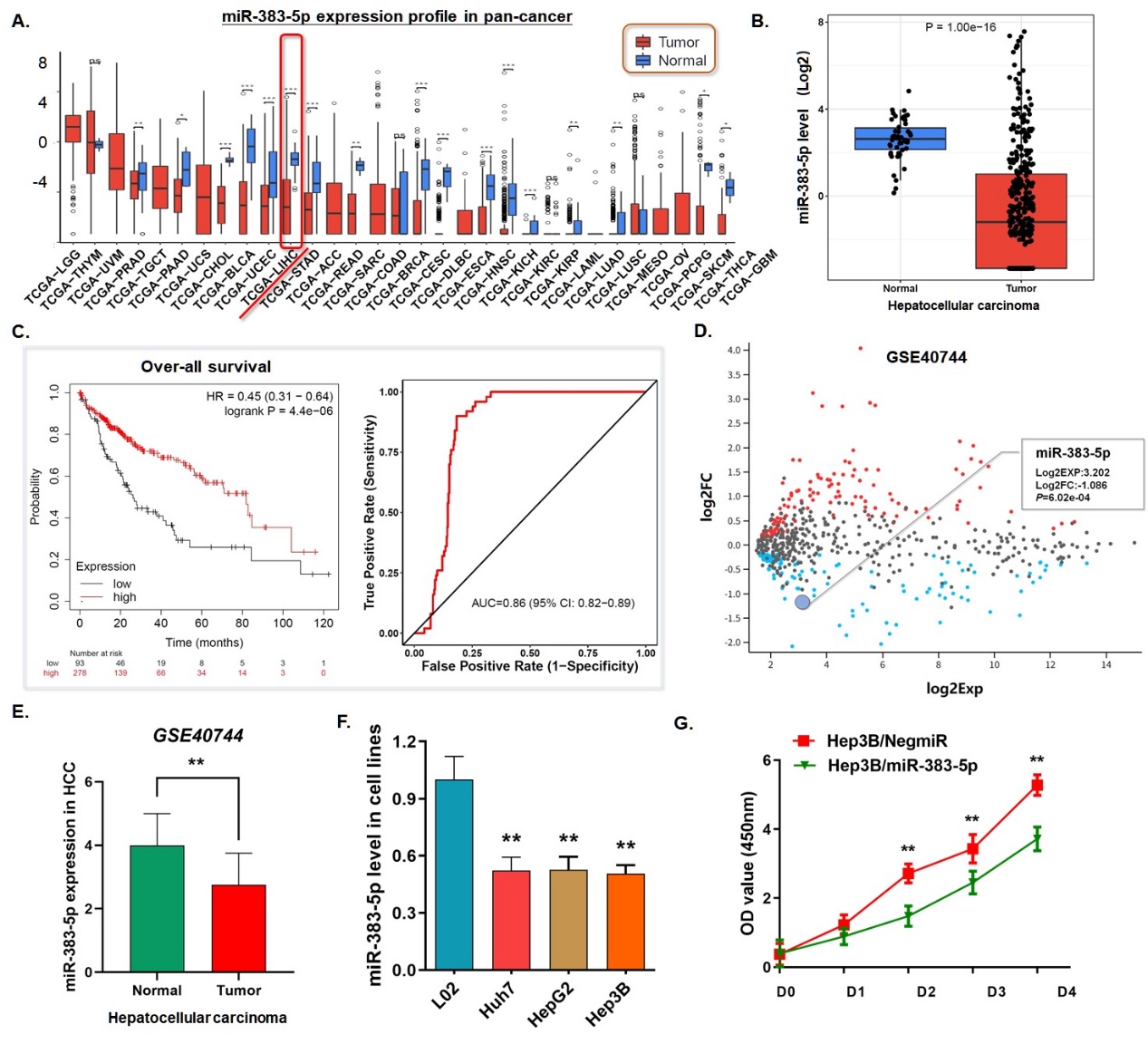


**Suppl. Fig. 2. MiR-383-5p expression profile and relationship with HCC cell proliferation**

**A.** The expression profile of miR-383-5p was calculated through the TCGA database. The expression levels of miR-383-5p in pan-cancer demonstrate a general down-regulation in HCC with high significance. **B.** The expression of miR-383-5p calculated from TCGA database information demonstrated a remarkable decrease in tumor tissues (*P*=1.0e-16). C. The defection of miR-383-5p is correlated with short overall survival (OS) in HCC patients (*P*=4.4e-06, HR=0.45). According to the ROC curve, miR-383-3p is confident in predicting the outcome of the HCC patients (AUC=0.85; 95%CI:0.82-0.89). **D～E.** The expression microRNA levels in HCC specimens were calculated by analyzing the GSE40744 dataset from the GEO database. The generated volcanic map demonstrates either the highly or lowly expressed microRNAs with significance. **F.** RT-qPCR assay demonstrated that miR-383-5p is significantly decreased in HCC lines compared with the LO2 cells as control. **G.** MiR-383-5p was introduced through the mimics method in Hep3B cells. The CCK8 assay indicated a significant suppression of cell proliferation ability by ectopically expression miR-383-5p (***P*＜0.001).
